# Five Years of Access and Activism

**DOI:** 10.1371/journal.pmed.1000167

**Published:** 2009-10-20

**Authors:** The PLoS Medicine Editors

## Abstract

*PLoS Medicine* announced a “refocusing of the journal's priorities” in April 2009, and the editors now look back over the last six months to assess the impact of the refocused scope.

In April 2009, we marked the five year anniversary *of PLoS Medicine*'s first call for papers with an editorial titled “A Medical Journal for the World's Health Priorities” [1]. The editorial was a renewed and revitalized call for papers, announcing a “refocusing of the journal's priorities.” Going forward, we said, we would prioritize papers addressing those diseases with the greatest global burden. We would also aim to be as broad a journal as possible, publishing papers that explored not just biological causes of illness, but also social, environmental, and political determinants of health. Six months later, as we now mark the journal's official five-year anniversary (our launch issue was October 19, 2004), has our refocused scope had any impact on what we publish?

Gro Harlem Brundtland, former director general of the World Health Organization, has argued that health problems are “no longer just local, national or regional, they are global” [2]. Events over the past six months support this view, and have reaffirmed to us that our new direction — unreservedly global and inclusive of politics, society, and the environment — makes sense. Pandemic H1N1 influenza (“swine flu”) was identified in April 2009 and has spread rapidly across the globe. In May 2009, the global health impact of climate change moved higher up the international agenda through the launch of an innovative multidisciplinary report by University College London and *The Lancet*, called “Managing the Health Effects of Climate Change” [3]. Throughout the summer, violent political conflicts flared up in many of the world's hotspots for war, such as Sri Lanka, Afghanistan, and the Democratic Republic of the Congo, causing major morbidity and mortality. These events illustrate how “human health remains inextricably intertwined with the environment — in its widest sense — in which we live,” as we wrote in our April 2009 editorial [1].

Authors have clearly taken notice of our refocused scope. We are now seeing an increasing number of cover letters that state that the accompanying submission is a response to our recent call for papers. Our new direction is also reflected in the papers that we have published in the last six months. Our focus on the global burden of disease can be seen in an international study, based on mental health surveys worldwide, examining the link between mental disorders and suicide [4]; in two systematic reviews that helped to answer important questions about drug treatment for tuberculosis [5],[6]; and in a randomized controlled trial of solar drinking water disinfection that showed it not to reduce childhood diarrhea despite widespread promotion of the intervention [7]. Our focus on the interconnectedness between health and the broader contexts (ecological, social, and political) is seen in a study of the impact of demographic transition on dengue [8]; in essays on the link between home foreclosures (repossessions) and public health [9] and on the health impact of Somalia's civil war [10]; and in our recently launched series on treating mental health problems in low- and middle-income countries [11].

We will, of course, continue to publish important laboratory and clinical studies that have clear implications for clinical medicine or public health. Examples of such studies from the last six months include the discovery and characterization of a new tumor suppressor gene, *ductal epithelium–associated RING Chromosome 1* (*DEAR1*) [12]; a cohort study showing that preconceptional folate supplementation is associated with a 50%–70% reduction in the incidence of early spontaneous preterm birth [13]; and a study on the preclinical natural history of serous ovarian cancer [14].

In addition to *PLoS Medicine* taking a new direction, PLoS spent the spring and summer engaged in four new projects and initiatives that highlight some of our underlying values, ideals, and commitments. First, *PLoS Medicine* is deeply committed to promoting transparency in medical research, which is why the journal, represented by the public interest law firm Public Justice (http://www.tlpj.org), intervened in an ongoing court case in which women were suing Wyeth, the manufacturers of Prempro, a hormone replacement therapy [15]. We intervened in the case in order to unseal a massive amount of documentary evidence showing a coordinated campaign of ghostwriting from Wyeth on articles about this drug and other hormone replacement therapies. Our intervention was successful, and on August 21, 2009 we created the “Wyeth Ghostwriting Archive,” which makes about 1,500 relevant documents publicly available [16].

Second, PLoS is continually working to refine and improve the communication of health and science research. The 2009 H1N1 influenza pandemic highlights the need for a new way for scientists to rapidly exchange data, and on August 20, 2009 PLoS responded by launching an experimental online platform called *PLoS Currents: Influenza* [17]. Submissions are assessed by an expert group of influenza researchers, but in the interest of timeliness they do not undergo in-depth peer review. Published articles are also deposited into a new independent research database run by the National Institutes of Health, called Rapid Research Notes [18]. A key component of the *PLoS Currents: Influenza* platform is a Google project called Google Knol, which allows community interaction, comment, and discussion around “knols” or units of knowledge [19]. Articles in *PLoS Currents: Influenza* will generally report preliminary findings, and authors are likely to develop their studies further before formally submitting them to a peer-reviewed journal (such as the PLoS journals).

Third, we have long been working on finding better ways to evaluate the impact of research. The impact factor assigned to a journal by Thomson Scientific is an extremely poor — and highly unscientific — measure of the worth of an individual study [20]. In June 2006, a *PLoS Medicine* editorial noted that “The opening up of the literature means that better ways of assessing papers and journals are coming — and we should embrace them” [20]. On September 15, 2009, PLoS took a big step toward better assessment by launching a set of metrics attached to every paper published in every PLoS journal that tells you, at a glance, what kind of impact a paper has had. The metrics include online usage data (page views and downloads), citation counts, comments and ratings by readers, social bookmarks, and blog coverage. As far as we know, this is the first attempt by a large publisher to publicly provide such a broad range of transparent usage data for each of its articles.

Finally, we believe that an important advantage of open access publication is the opportunity for engaging readers worldwide in an ongoing conversation about health and health research. On May 21, 2009 we launched a new venture aimed at such engagement, the *Speaking of Medicine* community blog (http://speakingofmedicine.plos.org/). Here you'll find a diverse array of blogs and podcasts from the *PLoS Medicine* editors and publications staff, members of the journal's editorial board, medical students, and invited “guest contributors.” Encouraged by reader comments, we have been posting new entries at an accelerating rate, and we now include regular features such as the “Daily Click” (a “pick of the day” from a member of the *PLoS Medicine* team, highlighting a particularly compelling item on the web) and links to relevant videos, pubcasts, and the PLoS Twitter feed (http://twitter.com/PLOS). We invite you to join the conversation.

In a 2005 article in the *Medical Journal of Australia*, Richard Smith, former editor of the *BMJ* and a current member of the PLoS Board of Directors, posed the question: “Can medical journals lead or must they follow?” [21]. He concluded that the main contribution of journals “may be less to try and achieve precise reform and more to put issues firmly on the agenda.” In our first five years, *PLoS Medicine* tried to show such leadership by demonstrating why open access matters to medicine, by working to break the cycle of dependency between medical journals and the marketing programs of drug companies, by raising standards of research reporting, and by making the case that a modern medical journal should look outward—beyond biology—and respond to the needs of a wider, global society. We look forward to engaging with whatever the next five years bring.
